# Editorial: Cellular and molecular responses to changes in nutrition and exercise

**DOI:** 10.3389/fncel.2022.1102308

**Published:** 2022-12-06

**Authors:** Oliver von Bohlen und Halbach

**Affiliations:** Institut für Anatomie und Zellbiologie, Universitätsmedizin Greifswald, Greifswald, Germany

**Keywords:** exercise, neuronal plasticity, synaptic plasticity, adult neurogenesis, hippocampus, serotonin

Neurological and neuropsychiatric disorders pose a significant burden on human health and society throughout the world. In the past 30 years, the absolute numbers of deaths and people with disabilities owing to neurological diseases have risen substantially (Feigin et al., 2020). The prevalence of overweight and obesity has also increased in the last decades worldwide (Ng et al., 2014). In this context it is important to note that obesity has been associated with a higher risk for developing neuropsychiatric disorders. For example, in a recent Swedish study, about half of all young people treated for severe obesity have neuropsychiatric problems (Bjork et al., 2021). In addition, obesity has been shown to be associated with declines in cognitive performance in humans. Thus, a higher body mass index (BMI) is associated with lower cognitive scores and a higher BMI at baseline is associated with a higher cognitive decline at follow-up (Cournot et al., 2006). Leptin-deficient (ob/ob; an animal model of obesity) mice display altered adult hippocampal neurogenesis (Bracke et al., 2019). In addition, leptin-receptor deficient (db/db, a further animal model of obesity) mice also show impairments in adult hippocampal neurogenesis (Ramos-Rodriguez et al., 2014) and disturbances in cognitive functions (Ramos-Rodriguez et al., 2013).

Dietary restriction can help reduce excess adipose tissue and obesity. Moreover, dietary restriction can increase life span in a wide variety of species, and may also promote neuronal survival. Various studies hint that nutrition has an impact on brain functions. Among others, dietary restriction has beneficial effects on neuronal plasticity, adult hippocampal neurogenesis and cognitive functions. Increasing energy expenditure can also help to reduce or balance body weight. Energy expenditure can, among others, be increased by increasing physical activity. Physical exercise is thought to play an important role in the prevention and delayed progression of neurological disease through a variety of cellular and molecular mechanisms. Indeed, an association between physical activity and mental health has been reported (see for details: Biddle and Asare, 2011; Lubans et al., 2016). Physical exercise, especially running, has effects on the brain morphology, including increased hippocampal volume (Biedermann et al., 2016). Exercise has profound effects on adult hippocampal neurogenesis and hippocampus-dependent learning (van Praag et al., 1999). Several molecular systems seemed to be important for maintaining neuronal function and plasticity in this context. Among these factors, the neurotrophins, especially, brain-derived neurotrophic factor (BDNF) plays a prominent role (Vivar et al., 2013). Mice deficient for BDNF indeed show altered hippocampal functions (von Bohlen und Halbach, 2010) and are getting obese over time (Kernie et al., 2000). Likewise, in humans, polymorphism in the BDNF gene (Val66Met) has been associated with obesity (Skledar et al., 2012). Moreover, an association of the Val66Met BDNF polymorphism with reduced hippocampal volumes in major depression has been reported (Frodl et al., 2007). Thus, nutrition and exercise can have a strong impact upon neuronal functions and neuronal plasticity. Whilst there is a growing body of work studying the effects of energy metabolism on the functions of the brain, there are still many unanswered questions on the mechanisms that underlie these changes and their potential role in disease prevention.

Two articles report about further molecules that are involved in regulating physical exercise stimulated adult hippocampal neurogenesis, namely the cyclin-dependent kinase inhibitor p16Ink4a (Micheli et al.) and adiponectin (Wang et al.). Ge and Dai report about the effects of 3-week treadmill exercise on the electrophysiological and channel properties of serotonergic neurons located in the dorsal raphe nucleus. In the paper by Liśkiewicz et al. it is analyzed whether stimulation of autophagy is one of the mediators of ketogenic diet induced neuroprotection in the hippocampus.

This Research Topic aimed to bring about new findings on the cellular and molecular processes that impact brain function in health and disease. The data presented here mainly focus on the beneficial role of ketonic diet and physical exercise on brain function, including the effects on neuronal circuitries and neuronal plasticity. Taken together, the articles support the view that the balance between nutrition and exercise is of paramount importance for cognitive health and the maintenance of brain structures.

## Author contributions

The author confirms being the sole contributor of this work and has approved it for publication.
